# Vicarious trauma on the hemodialysis healthcare workers in the besieged Ethiopia’s Tigray region: a call to action

**DOI:** 10.1186/s12916-022-02637-1

**Published:** 2022-11-08

**Authors:** Ephrem Berhe, Bisrat Tesfay, Hale Teka

**Affiliations:** 1 Ayder Comprehensive Specialized Hospital, College of Health Sciences, Mekelle University, Mek’ele, Tigray Ethiopia; 2 Ayder Comprehensive Specialized Hospital, Department of Internal Medicine, Nephrology Unit, Mekelle, Ethiopia

**Keywords:** Hemodialysis, Healthcare workers, Vicarious trauma, Tigray, War, Siege, Ethiopia

## Abstract

**Background:**

The war on Ethiopia’s Tigray broke out on November 4, 2020. Amid the armed conflict, governmental institutions were destroyed, people were displaced, and thousands of civilians were killed. The region was experiencing an on-and-off type of blockade since the war broke out until June 28, 2021, at which time the federal government of Ethiopia imposed a siege cutting off the region from the rest of the world. Due to the shortage of medicines and medical supplies, witnessing deaths that otherwise were preventable under normal conditions has become the daily predicament of healthcare workers. The burden of healthcare disintegration is particularly carried by patients with chronic medical illnesses including patients on dialysis.

**Main body:**

Ayder hospital, Tigray’s flagship healthcare institution, hosts the only hemodialysis center in the entire region. This center is currently unable to give appropriate care to kidney failure patients for a lack of access to dialysis supplies and consumables due to the ongoing war and siege. This has resulted in vicarious trauma manifested with compassion fatigue, irritability, a feeling of bystander guilt; sadness about the patient’s victimization, and hopelessness among healthcare workers caring for dialysis patients.

**Conclusion:**

The suffering of veteran patients and witnessing preventable deaths have continued to haunt and torment healthcare workers in the dialysis unit leading to vicarious trauma. Cognizant of the fact that vicarious trauma has serious health ramifications on healthcare workers; we call up the international community to advocate for a full resumption of access to healthcare and the provision of mental health support and educate and train healthcare workers dealing with end-stage kidney disease patients on hemodialysis.

## Background

Tigray is a region in northern Ethiopia, with an estimated population of over 7 million [1]. A tragic and brutal civil war has been waging in Tigray since November 2020. During the active fighting, more than 70% of healthcare facilities had been either deliberately vandalized or looted [2]. The region was experiencing an on-and-off type of blockade since the war broke out. Eight months into the war, the Ethiopian federal government imposed a siege on the war-wracked region after the Tigrayan forces recaptured the capital Mekelle in June 2021. Except for trickles of some medications from international donors, almost no medical supply has made it to the region adding strain to the already fragmented healthcare system [3]. This has no equivalency other than the denial of the fundamental human right to access healthcare services [4]. The burden of healthcare disintegration is mainly carried by patients with chronic medical illnesses including patients on hemodialysis (HD). A full-scale war and siege can have shattering consequences for patients requiring constant care and well-functioning health infrastructures, particularly in resource-limited settings where delivering optimum care are already challenging [5]. Hemodialysis necessitates substantial quantities of energy, water, and reliable delivery of a broad array of supplies and therapeutics. The ongoing war and siege have put a burden on all these requirements, generating a myriad of medical and logistic problems and inflicting the lives of patients at a greater risk.

## Main text

Ayder hospital is the only hospital offering HD services in the entire region [6]. Like other hospitals in the region, this hospital has been severely affected by the ongoing war and siege. The HD center which is established as a public partnership model almost a decade ago is particularly severed. Dialysis utilization has been drastically curtailed as patient enrollments in the dialysis center have decreased from 110 in 2020 (before the war) to 69 in 2021(after the war) [7]. Currently, until September 2022, there were 25 patients under suboptimal HD. Patients with otherwise treatable kidney failure are dying. Overall, the percentage of mortality in patients receiving HD has doubled from 25.5% before the war to 53.1% after the war broke out [7]. The severe symptoms of suffering patients and the inevitable preventable death in front of the attending healthcare workers lead to vicarious trauma.

The dialysis unit represents a unique population of end-stage kidney disease (ESKD) distinguished by a significant burden of disease and high mortality rates [8]. Amid the war and siege, due to the dearth of dialysis supplies and consumables, Ayder’s HD unit cannot give appropriate care [7]. Despite heroic but dangerous improvisations, the hospital’s dialysis service has collapsed putting many lives with otherwise treatable kidney problems at stake. The challenges faced by the HD center of this hospital are unprecedented. Patients with ESKD in Tigray can neither receive optimal HD nor can be referred to the capital city of Ethiopia for renal replacement therapy [9].

## Who cares for the healthcare workers?

Healthcare workers in the HD unit are expected to be fully present to give focused attention to patients while attending to their emotional and physical needs of the patients. With the added burden of responsibility to give care under compromised healthcare services amid the war and siege, witnessing the sufferings and death of the veterans due to lack of optimum HD is the daily predicament of the dialysis care team. As such, the dialysis team is expected to meet the permeative health requirement of the sick in the era of dynamic healthcare. This subjects healthcare workers to different spectrums of emotional trauma as a result of continuous exposure to patient symptoms. They tend to be too sympathetic to the harrowing day-to-day experience of their ill-fated clients leading to a blurring of professional boundaries. As patients with ESKD face a great deal of physical and mental misery, the healthcare workers in the HD unit are particularly vulnerable bearing witness to the devastating impact of this manmade catastrophe. Healthcare workers exposed to such stressful environments will eventually develop exhaustion; fail to be compassionate to their patients, and ultimately fail to perform at their best level [10].

## The hidden danger: vicarious trauma in healthcare workers

**Fig. 1:**
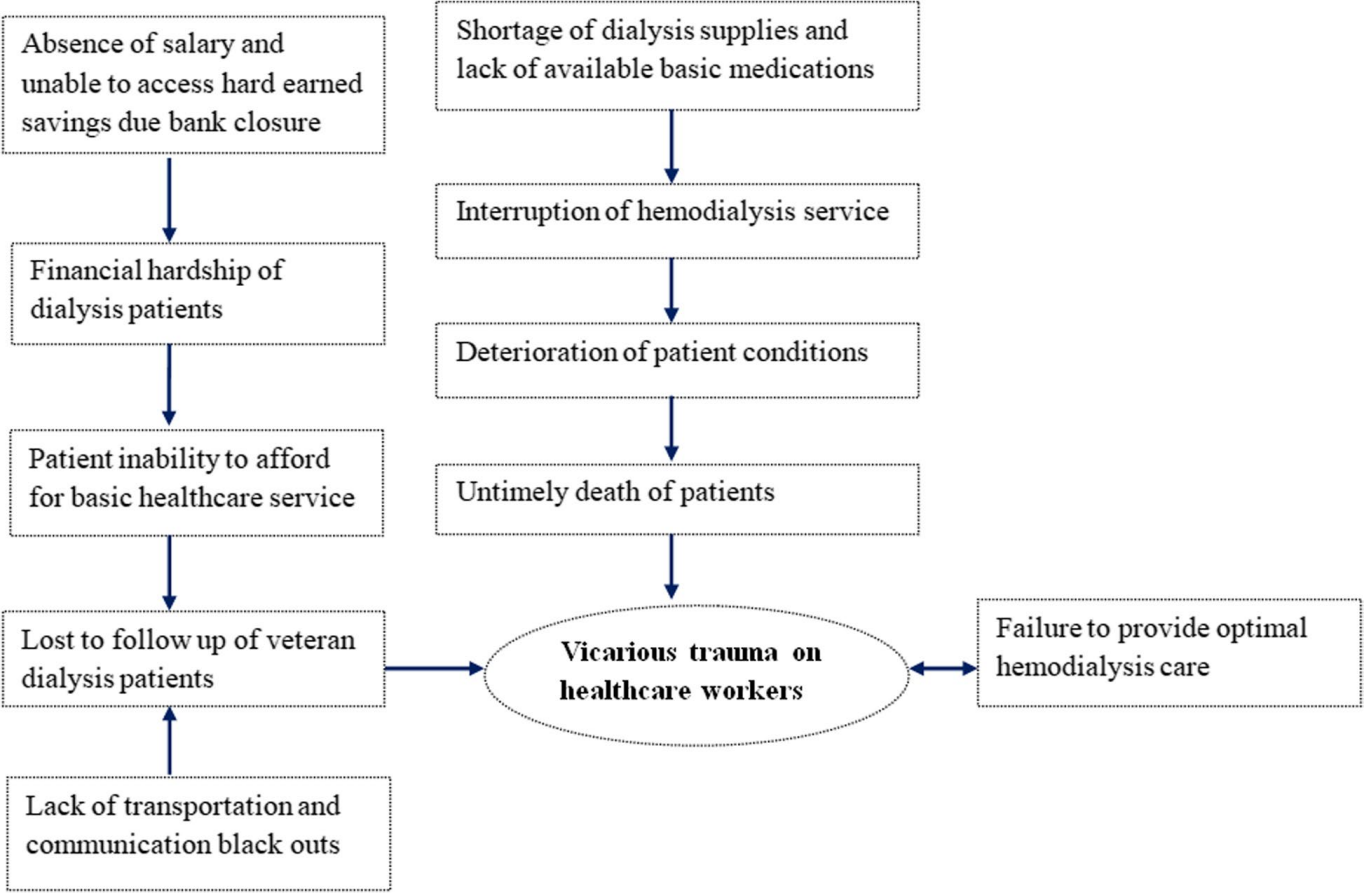
Conceptual framework of vicarious trauma among healthcare workers in the hemodialysis unit of Ayder hospital, Tigray, northern Ethiopia

Significant time exposure and empathetically listening to the stories of suffering and traumatic complaints from suboptimal dialyzed ESKD patients is made worse as the healthcare workers are unable or insufficiently resourced to help. Inadequate supervision and support of the organizational structures and the lack of opportunities to speak out due to the restricted access to communication are among the multipronged work-related factors of vicarious trauma of the healthcare workers in the HD center. As a result, hopelessness, physical signs of poor sleep, aches, pains, and illnesses, and feeling of insecurity and vulnerability are the other signs and symptoms the healthcare workers have manifested. Moreover, overly emotional involvement with patients and being preoccupied with the thoughts of patients outside of the work environment have become rampant. The guilt of failure to deliver appropriate care due to lack of service amid the conflict and siege further fuels the vicarious trauma (Fig. 1).

The different aspects of loss experienced in the HD unit daily further perturb the work environment and impair the quality of life and coping mechanisms of the healthcare workers. Over time, this emotional drainage can make the professionals develop low self-esteem, emotional numbing, and hate the drudgery of their work especially when there is no attention paid to such happenings.

When the suffering becomes out of control, healthcare workers usually look for solutions from their colleagues and the administration. However, the working environment is not supportive and empowering as trauma is ubiquitous during the ongoing war and siege. Expected to fulfill the colossal work of giving patient-focused complex care during this trying time, healthcare workers in the HD unit find it difficult to get a supportive and safe work environment. Facility leaders usually tend to overlook and fail to respond to the vicarious trauma of their healthcare workers as are caught with the more obvious financial and supply hurdles of the hospital even though both problems equally compromise service delivery.

## Conclusions

Healthcare workers in the dialysis center get emotionally entrapped in the constant suffering of their patients during war and siege. Unable to bear the vicarious trauma, the healthcare workers develop dysfunctional coping mechanisms which affect patient care as they become weary of the continuous pain. Not giving due care to patients further creates additional sympathetic trauma. This level of burnout results in a negative atmosphere in the unit and dissatisfaction at work. Hence, vicarious trauma begets vicarious trauma. Despite the adverse impacts of vicarious trauma will have on service delivery, facilities do not usually give enough attention to it as they focus on the more visible effects of the war and siege.

## Way forward

Lack of access to life-saving services is emotionally disturbing for healthcare workers, who know that patients are unlikely to receive the adequate assistance and support they require. Cognizant of the fact that vicarious trauma has serious health ramifications on healthcare workers, we call upon global researchers, policymakers, and the international community: firstly is to advocate for a full resumption of access to health to the millions of civilians caught in this tragic war in general and patients on dialysis in particular; secondly, the provision of mental health support and education to healthcare workers who are at immediate risk of post-traumatic stress disorder, anxiety, and other stress-related conditions in such drastic situations cannot be over-emphasized; thirdly, organizations must pay particular attention to healthcare workers, who handle chronically ill patients including patients on dialysis and who are prone to sustain vicarious trauma; and fourthly, the United Nations agencies, international physician societies, and independent national and international organizations should press all parties in this conflict to adhere to the international conventions and laws and respect human rights including the right to access healthcare.

## Data Availability

All relevant data are within the manuscript file.

## Abbreviations

ESKD: End-stage kidney disease
HD: Hemodialysis
